# Zilver PTX Post-market Surveillance Study of Paclitaxel-Eluting Stents for Treating Femoropopliteal Artery Disease in Japan: 2-Year Results

**DOI:** 10.1007/s00270-018-2110-1

**Published:** 2018-11-08

**Authors:** Kimihiko Kichikawa, Shigeo Ichihashi, Hiroyoshi Yokoi, Takao Ohki, Masato Nakamura, Kimihiro Komori, Shinsuke Nanto, Erin E. O’Leary, Aaron E. Lottes, Scott A. Snyder, Michael D. Dake

**Affiliations:** 1 Department of Radiology, Nara Medical University, Kashihara, Japan; 2 Department of Cardiovascular Medicine, Fukuoka Sanno Hospital, Fukuoka, Japan; 3 Department of Surgery, Jikei University Hospital, Tokyo, Japan; 4 Division of Cardiovascular Medicine, Ohashi Medical Center, Toho University, Tokyo, Japan; 5 Division of Vascular Surgery, Division of Surgery, Nagoya University Graduate School of Medicine, Nagoya, Japan; 6 Nishinomiya Hospital Affairs, Nishinomiya Municipal Central Hospital, Nishinomiya, Japan; 7 Cook Research Incorporated, West Lafayette, IN USA; 8 Department of Cardiothoracic Surgery, Stanford University Medical Center, Stanford, CA USA

**Keywords:** Drug eluting stent, Paclitaxel-eluting stent, Peripheral artery disease, Femoropopliteal artery

## Abstract

**Purpose:**

A prospective, multicenter post-market surveillance study in Japan evaluated the 2-year safety and effectiveness of the DES in real-world patients with complex femoropopliteal artery lesions.

**Methods:**

There were no exclusion criteria, and consecutive symptomatic patients with femoropopliteal lesions treated with the DES were enrolled in the study. Clinically driven target lesion revascularization (TLR) was defined as reintervention performed for > 50% diameter stenosis after recurrent clinical symptoms of peripheral arterial disease. Clinical benefit was defined as freedom from persistent or deteriorating ischemic symptoms. Patency was evaluated by duplex ultrasound where physicians considered this standard of care.

**Results:**

In this study, 905 patients were enrolled at 95 institutions in Japan. There were numerous comorbidities including a high incidence of diabetes (58.8%) and chronic kidney disease (43.6%). Additionally, 21.4% of patients were classified with critical limb ischemia. Lesions were complex, with an average length of 14.6 ± 9.6 cm (range 0.5–40 cm), 41.5% total occlusions, and 18.7% in-stent restenosis. In total, 1861 DES were placed in 1080 lesions. Two-year follow-up was obtained for > 90% of eligible patients. Freedom from TLR was 83.7%, and clinical benefit was 80.0% through 2 years. The 2-year primary patency rate was 70.3%. Rutherford classification significantly improved (*p *< 0.01), with approximately 80% of patients classified as Rutherford class 0 or 1 at 2 years.

**Conclusion:**

Despite more challenging lesion characteristics, 2-year results from the current study are similar to outcomes from the previous Zilver PTX studies, confirming the efficacy of the Zilver PTX DES in a complicated femoropopliteal lesion (Zilver PTX Post-Market Study in Japan; NCT02254837).

**Level of Evidence:**

Post-market surveillance study, Level III.

## Introduction

Endovascular therapy with balloon angioplasty and/or stent placement is a standard treatment for peripheral arterial disease (PAD). Treatment strategy for femoropopliteal (FP) lesions is still controversial. A major limitation of bare metal stents (BMS) is high prevalence of intimal hyperplasia, especially in long lesions. The primary patency rate after BMS placement in the FP lesions has been reported to be 53–81%, 36–72%, 19–66% at 1, 2, 3 years [1–3], and these results highly vary depending on the lesion backgrounds. To combat this issue, Zilver PTX stent, a polymer-free, paclitaxel-coated nitinol-eluting stent (DES), is available for clinical use. There are several global clinical studies evaluating the efficacy of the DES including more than 2400 patients. Previously, the results from a large randomized controlled study (RCT) and a complementary, large single-arm study (SAS) supported the safety and effectiveness of the DES. Currently, a multicenter, prospective, post-market surveillance study (PMS) is underway in Japan to further evaluate this stent in real-world populations including longer and more complex lesions. One-year outcomes demonstrated the safety and effectiveness of the DES in patients with complex lesions [4]. Herein, 2-year outcomes of the study are reported.

## Methods

This prospective, multicenter study (Japan PMS) enrolled 905 consecutive Japanese patients with symptomatic PAD involving the above-the-knee femoropopliteal arteries who were treated with the Zilver PTX Drug-Eluting Peripheral Stent (Cook Medical, Bloomington, IN). Detailed descriptions of study design, methods, and follow-up through 1 year for the study were previously reported [4]. Notably, inclusion of all patients treated with the device was planned, and there were no exclusion criteria. This study was regulated by the Japanese Ministry of Health, Labour, and Welfare and was therefore required to be conducted in accordance with Japanese Good Post-market Surveillance Practice Regulations, which dictate that informed consent processes to be determined by each institution’s ethical committee policy to specify whether informed consent was necessary or outcome data could be abstracted while protecting patient’s rights without requiring individual patient consent.

### Interventions and Medications

Device instructions for use recommend that stents be oversized by 1–2 mm with respect to the reference vessel and placed at least 1 cm below the SFA origin and above the medial femoral epicondyle. Treatment of lesions in both legs was permitted, and multiple lesions per limb were also allowed. Pre-dilatation of the lesion, post-dilatation of the stent, and treatment of inflow and outflow disease were at the physician’s discretion. The same antiplatelet regimen used in previous studies was recommended for all patients: clopidogrel or ticlopidine starting at least 24 h prior to the procedure, or a procedural loading dose; continued clopidogrel or ticlopidine therapy for at least 60 days post-procedure; and aspirin indefinitely.

### Follow-Up Assessment

Clinically driven target lesion revascularization (TLR) was defined as reintervention performed for ≥ 50% diameter stenosis within ± 5 mm of the target lesion after recurrent clinical symptoms of PAD. Thrombosis was site-reported as total occlusion of suspected thrombotic origin. Clinical benefit was defined as freedom from persistent or worsening symptoms of ischemia (i.e., persistent or worsening claudication, rest pain, worsening Rutherford class, ulcer, tissue loss, or other symptoms indicating the need for reintervention) after the initial study treatment. ABI and Rutherford classification were assessed pre-procedure, prior to discharge, and at the 1- and 2-year clinical visits. Patency was evaluated by duplex ultrasonography at institutions where physicians considered this standard of care, with loss of patency corresponding to a peak systolic velocity ratio ≥ 2.4. Stent integrity was assessed by radiography at 1 year, with the next evaluations at 3 and 5 years. Deaths were adjudicated by an independent clinical events committee.

### Statistical Analysis

As previously described [4], a sample size of 900 was selected to provide 95% confidence for determination of events at rates as low as 1–2%. The data were analyzed using SAS 9.3 (SAS Institute Inc., Cary, NC). Continuous variables were summarized with means and standard deviations, with *p* values calculated using the standard *t* test. Dichotomous and polytomous variables were reported as counts and percentages, with *p* values calculated using Fisher’s exact test. As appropriate, the number of observations represented the number of patients or the number of treated lesions. Kaplan–Meier analyses were performed to assess freedom from TLR, freedom from thrombosis, clinical benefit, and patency over time.

## Results

Demographics, comorbidities, and baseline lesion characteristics reported by the investigative sites are shown in Table 1. There were numerous comorbidities including a high incidence of diabetes (58.8%) and chronic kidney disease (43.6%). Additionally, 21.4% of patients were classified with critical limb ischemia. The lesions were complex, with an average lesion length of 14.6 ± 9.6 cm (range 0.5–40 cm), 41.5% total occlusions, and 18.7% in-stent restenosis (ISR).

**Table 1:** Baseline demographics and lesion characteristics

*Patient characteristics*	
Patients	905
Mean age (years)	73.5 ± 8.5 (905)
Men	70.3 (636)
Diabetes	58.8 (532)
Hypertension	85.4 (773)
Hypercholesterolemia	60.8 (550)
Chronic kidney disease	43.6 (395)
eGFR < 60 mL/min/1.73 m^2^ and/or dialysis	35.5 (321)
Pulmonary disease	8.1 (73)
*Lesion characteristics*	
Lesions	1080
Lesion length (cm)	14.6 ± 9.6 (1079)
Lesions > 15 cm	41.9 (453)
Lesions > 20 cm	29.7 (321)
Lesion location^a^	
Proximal SFA	61.1 (657)
Distal SFA	64.5 (693)
Popliteal	9.4 (101)
Total occlusion	41.5 (448)
In-stent restenosis	18.7 (202)
Percent diameter stenosis	91.7 ± 10.8 (1080)
Reference vessel diameter (mm)	5.7 ± 0.9 (1079)
Critical limb ischemia (Rutherford classes 4–6)^b^	21.4 (218)
Number of patent runoff vessels^c^	
0	6.6 (71)
1	31.9 (343)
2	32.5 (349)
≥ 3	29.0 (311)

Values are mean ± SD or % (*n*)

*eGFR* estimated glomerular filtration rate, *SFA* superficial femoral artery

^a^Of the 1080 lesions in this study, 376 lesions span more than one segment

^b^Rutherford classification data not available for 60 lesions

^c^Data not available for six lesions

Two-year clinical follow-up data were available for 662 patients, which represents > 90% of the 709 patients eligible for 2-year follow-up. All-cause mortality was 10.5% through 2 years. There were no device-related deaths. Additionally, 25 patients withdrew and 76 patients were lost to follow-up prior to the 2-year visit. No paclitaxel-related adverse events were reported. Ten patients in the study had an amputation for a 2-year rate of 1.1%. Of these 10 amputations, eight patients were classified as Rutherford 5 pre-procedure.

**Fig. 1:**
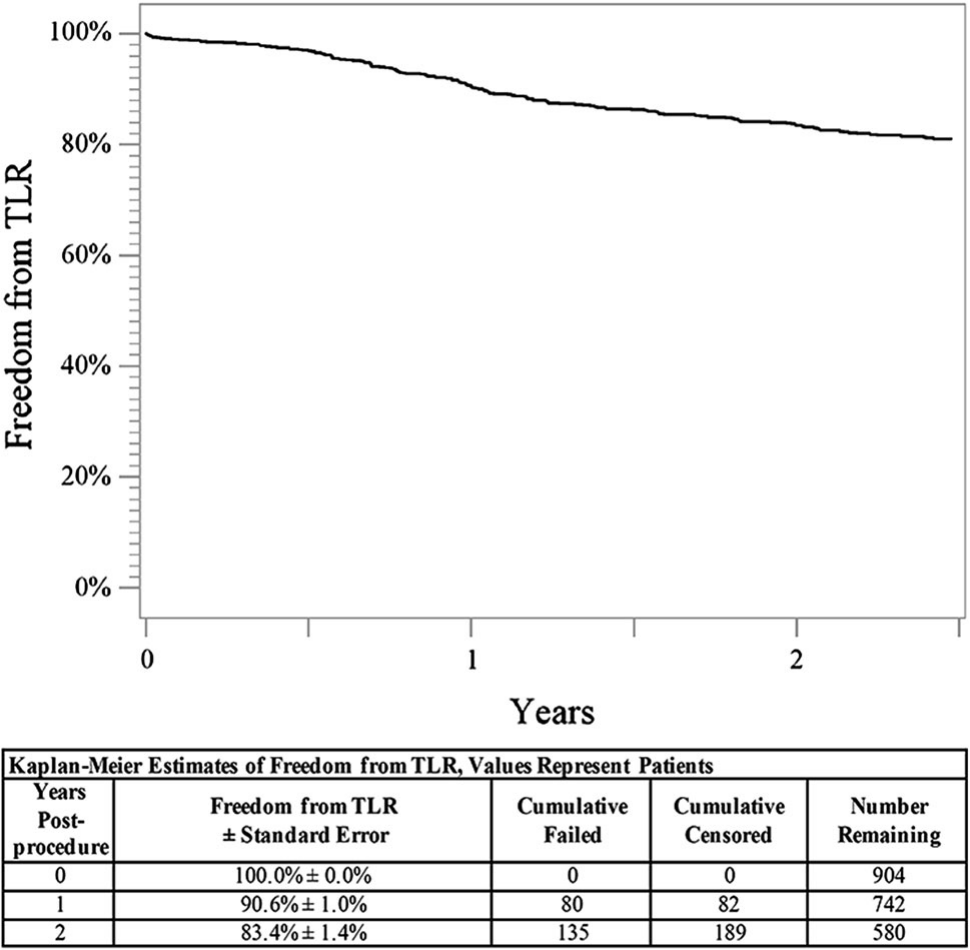
2-Year freedom from TLR. The Kaplan–Meier curve shows 83.7% freedom from TLR through 2 years for patients treated with the DES. The life table is included. *DES* drug-eluting stent, *TLR* target lesion revascularization

**Fig. 2:**
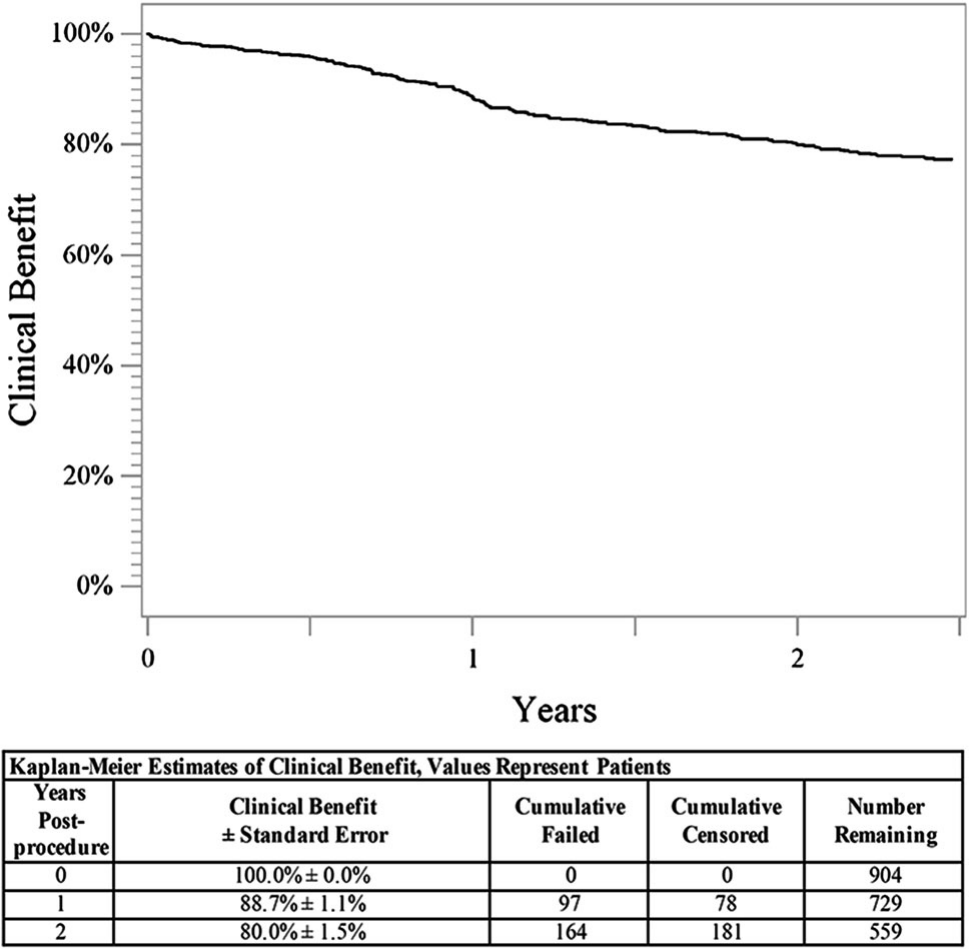
2-Year clinical benefit. The Kaplan–Meier curve shows 80.0% clinical benefit through 2 years for patients treated with the DES. The life table is included. *DES* drug-eluting stent

**Fig. 3:**
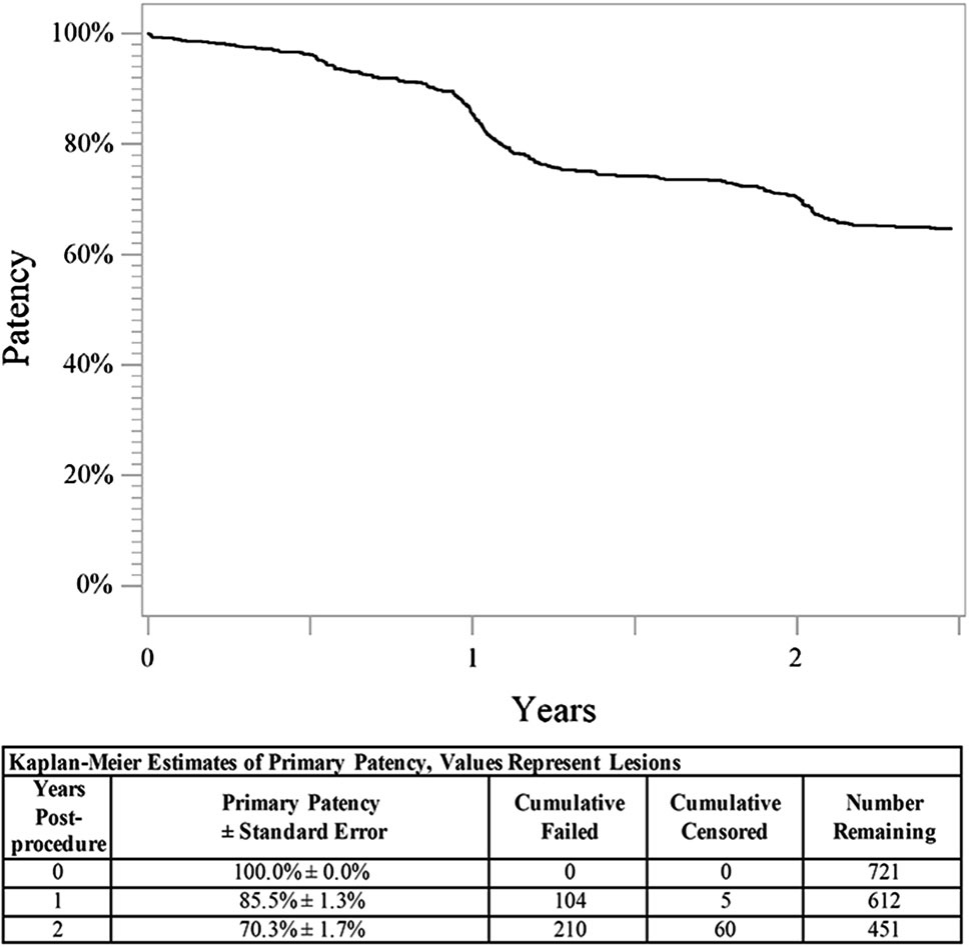
2-Year primary patency. The Kaplan–Meier curve shows 70.3% primary patency through 2 years for lesions treated with the DES. The life table is included. *DES* drug-eluting stent

Through 2 years, the Kaplan–Meier estimate of freedom from clinically driven TLR was 83.7% (Fig. 1), and the freedom from thrombosis rate was 95.9%. Clinical benefit, defined as freedom from persistent or worsening symptoms of ischemia (i.e., claudication, rest pain, ulcer, or tissue loss), was 80.0% at 2 years (Fig. 2). Clinical assessment at 2 years also revealed a significant improvement in Rutherford classification compared to baseline (*p *< 0.01) (Table 2). Clinical improvement of at least one Rutherford class was achieved in 563 of 654 patients (86.1%) through 2 years. Approximately 80% of patients were classified as Rutherford class 0 or 1 at 2 years, and the median Rutherford classification improved from class 3 to class 0 (Table 2). Similarly, the mean ABI significantly improved from baseline through 2 years from 0.63 to 0.85 (*p *< 0.01) (Table 2). Patency was assessed by ultrasound when it was standard of care and is reported for 58% of patients. Based on Kaplan–Meier estimates, the 2-year primary patency rate was 70.3% (Fig. 3). There were no significant differences in demographics, lesion characteristics, TLR rate, or thrombosis rate between patients with and without ultrasound surveillance.

**Table 2:** Clinical outcomes

Clinical outcome	Pre-procedure	1-Year*	2-Years*
ABI	0.63 ± 0.18 (980)	0.86 ± 0.17 (826)	0.85 ± 0.18 (646)
Rutherford class^a^			
0	1.0 (10)	54.1% (429)	52.2% (358)
1	7.3% (74)	23.6% (187)	27.8% (191)
2	26.6% (271)	11.0% (87)	11.8% (81)
3	43.8% (447)	5.5% (44)	4.7% (32)
4	10.3% (105)	2.8% (22)	1.6% (11)
5	9.7% (99)	2.5% (20)	1.3% (9)
6	1.4% (14)	0.5% (4)	0.6% (4)

Values are mean ± SD or % (*n*)

*ABI* ankle brachial index

^a^Pre-procedure Rutherford class was obtained for 1020 lesions, 1-year Rutherford scores were obtained for 793 lesions, and 2-year Rutherford scores were obtained for 686 lesions

*Statistically significant compared to pre-procedure, *p *< 0.01

## Discussion

The DES has been studied in multiple clinical investigations including a RCT comparing to PTA and BMS, a complementary large single-arm study (SAS), and a number of registries [4–8]. The Zilver PTX RCT confirmed the superiority of the DES compared to both PTA and BMS through 5 years, demonstrating the durable effect of the paclitaxel-coated DES [7]. The Zilver PTX SAS supported these results in a wider range of lesions. The present study had no exclusion criteria and therefore represented a real-world patient population: the average lesion length was 14.6 cm with approximately 30% of the lesions > 20 cm in length, 40% had only one or no vessel runoff, and a high rate of total occlusions and in-stent restenosis was documented [4].

Despite the complex lesions enrolled in this study, the results compare favorably to previously reported results. Dake et al. [7] reported a clinical benefit rate of 82.0% through 2 years in the RCT, which is similar to the 2-year rate of 80.0% in this study. The 2-year freedom from TLR rate of 83.7% was also similar to the rate of 86.0% reported in the RCT [7]. The primary patency rate in the current study was 70.3% at 2 years, which also compares favorably to the 76.3% 2-year patency rate reported in the RCT [7], especially considering the real-world population in the current study. Positive results with the DES for patients with poor runoff vessels or chronic renal failure, which have been reported as risk factors for restenosis after BMS placement [2], are supportive of the DES usage in complicated situations [9, 10].

Historically, patency of TASC C/D FP lesions has not been satisfactory, including results reported for patients in Japan. In a comparison study of SMART and MISAGO BMS placed in Japanese patients, the study population was stratified by TASC classification. In propensity score-matched cohorts, the 2-year patency rates in TASC C/D FP lesions were 62% for the SMART group and 25% for the MISAGO group [11]. Aihara et al. [12] compared the clinical outcomes of endovascular treatment, which consisted of stent placement in 70% of cases, and bypass surgery performed for claudicants with TASC C/D FP lesions. The 2-year patency rates were 58.2% in the endovascular arm and 75.5% in the bypass arm. The current results with the DES from the Japan PMS compare favorably to those previously reported after implantation of BMS, including SMART and MISAGO stents, and also comparable to that of bypass surgery.

Previous studies have typically only reported thrombosis rates through 1 year. Angioscopic observations in Japan have reported some evidence of thrombus with possible incomplete endothelial coverage at 2 months, but adequate healing at 1 year [13, 14]. Based on these observations, the continuation of dual antiplatelet therapy could be important to minimize the incidence of stent thrombosis. In the ZEPHYR registry in Japan, the 1-year thrombosis rate was 2% [8], similar to the 1-year rate of 3.0% for this study. The OSPREY trial, a single-arm multinational study, evaluating the performance of the MISAGO BMS, reported a 1-year stent thrombosis rate of 0.4% [4, 15]. Additionally, a study in Japan of the GORE Viabahn stent graft reported a 2.9% occlusion rate through 1 year [16]. A large multicenter registry in the USA evaluated stent thrombosis rates for BMS, DES, and stent grafts and reported a 4.3% stent thrombosis rate over a median follow-up period of 6 months [17].

In the present study, the rate of stent thrombosis through 2 years remained low, with a 1.1% change from 1 to 2 years. The low rate of thrombotic occlusion through 2 years in the present study is comparable to previously published 1-year rates. Another important consideration is that stent thrombosis in the SFA can be difficult to distinguish from total occlusion caused by restenosis, and the possible inclusion of stent occlusions that are of restenotic rather than thrombotic origin may overestimate the DES thrombosis rate, making direct comparisons among studies difficult.

There are several limitations in the study. First, stent patency was assessed only on a subgroup of patients where physicians considered this standard of care, as previously discussed. Therefore, results in terms of patency should be carefully interpreted. Second, only Japanese ethnicity was investigated in the study and other ethnic groups should be evaluated in a future to validate the efficacy of the Zilver PTX in a real-world practice. However, in the previous RCT, the results with the DES in the Japanese subgroup showed no major differences in safety and effectiveness compared with the non-Japanese patient group [18]. Therefore, the current favorable results from the Japan PMS might be extrapolated to other races having severe lesion backgrounds. Finally, this was a post-market, single-arm study without an internal control group; however, the study enrolled a large number of patients with high follow-up rate. The outcomes obtained with the DES in this study are consistent with those from the previously published RCT and the complementary SAS.

In conclusion, 2-year Japan PMS results are positive, confirm the long-term benefit of the DES, and reaffirm safety and effectiveness of the DES in complicated FP lesions. Consistent results across studies provide added support for the established long-term performance of the DES.
